# Brain Network Correlates of Emotional Aging

**DOI:** 10.1038/s41598-017-15572-6

**Published:** 2017-11-14

**Authors:** Youngwook Lyoo, Sujung Yoon

**Affiliations:** 1 Seoul National University College of Medicine, Seoul, South Korea; 2 Ewha Brain Institute, Ewha Womans University, Seoul, South Korea

## Abstract

Physical and cognitive functions typically decline with aging while emotional stability is relatively conserved. The current proof-of-concept study is the first to report of the brain mechanisms underlying emotional aging from a brain network perspective. Two hundred eighty-six healthy subjects aged 20–65 were classified into three groups of the emotionally young, intermediate-aged, and old (E-young, E-intermediate, and E-old, respectively) based on the cluster analysis of the emotion recognition task data. As subjects get emotionally older, performance on happiness recognition improved, while that on recognition of negative emotions declined. On the brain network side, there was a significant linear decreasing trend in intra-network functional connectivity of the visual and sensorimotor networks with emotional aging (E-young > E-intermediate > E-old) as well as chronological aging (C-young > C-intermediate > C-old). Intra-network functional connectivity of the executive control network (ECN), however, steadily increased with emotional aging (E-young < E-intermediate < E-old) but not with chronological aging. Furthermore, the inter-network functional connections between the ECN and default mode network were also greater in the E-old group relative to the E-young group. This suggests that the top-down integration of self-referential information during emotional processing becomes stronger as people get emotionally older.

## Introduction

Mounting evidence suggests that emotional stability, unlike other physiological functions of the body that tend to deteriorate, is often well-conserved even with aging^1–3^. Previous studies have found that reactions to negative emotional stimuli is attenuated, while the amount of focus and attention on positive emotional state is increased in healthy aging^4^. This general agreement regarding the conservation of emotional stability despite aging suggests a paradox to our understanding of aging, as most physical and brain functions peak during the early twenties then continue to decline thereafter^5,6^.

It is indispensable to efficiently recognize facial emotional expressions for nonverbal social communication. Prior functional studies were conducted to identify various brain regions that are involved in emotion recognition in normal and pathological conditions^7–10^. These studies suggest that several brain regions are typically engaged in facial emotion recognition in a sequential manner according to the characteristics and salience of emotions^9^. Specifically, dynamic interactions between brain regions involved with perceptual processing of facial features and those involved with understanding the emotional meaning of facial expression are necessary for efficient recognition of emotional state^9^. As part of this process, information about motivational preference and self-referential information is also transferred between relevant brain regions, thus improving the accuracy of emotion recognition by providing additional subject-specific emotional meaning^9,10^. This implies that interactions of complex and hierarchical brain network, rather than simply being dependent on the function of individual neuroanatomical areas of the brain, is important in identifying facial emotion.

Although it is relatively well known that in healthy aging, perception and recognition of facial emotion seems to be directed more toward positive emotions and lesser on negative emotions^2,5^, no study has been performed to determine brain network correlates of these aging-related changes in emotional processing.

In this study, using the emotion recognition task (ERT) and resting-state functional brain magnetic resonance imaging (fMRI) data from 286 healthy subjects covering the adult age span (20–65 years old), we examined whether the intra-network functional connectivity of major resting-state networks (RSNs) differ according to the subjects’ emotional age. Given the potential inter-individual variabilities in the age-related trajectory in emotional processing and the absence of consensus regarding a fixed criterion for defining emotional age, we have classified subjects into three groups based on a cluster analysis of performance on the ERT. Considering that older adults may utilize the top-down control system in emotional processing more so than younger adults^2,11–13^, we hypothesized that the intra-network functional connectivity of RSNs, particularly the high-function brain networks, would change according to the level of emotional aging. Finally, given that RSNs can be dynamically linked to each other and that these interconnections subserve specific aspects of cognitions and behaviors^14^, we assessed emotional aging-related differences in inter-network functional connections by evaluating the temporal coupling between the spontaneous resting activity of network components.

## Results

### Empirically Derived Groups Based on Cluster Analysis

Percent correct scores for the recognition on six emotions from the ERT (happiness, sadness, surprise, anger, disgust, and fear) were used as input variables for cluster analysis. A three-group solution was yielded with a significant differential level and pattern of performance on the ERT with the hierarchical agglomerative cluster analysis followed by K-means cluster analysis. Discriminant function analysis with a leave-one-out cross validation showed that the three-clusters were correctly classified by the percent correct scores of each emotion with a prediction accuracy of 95.1%.

**Figure 1:**
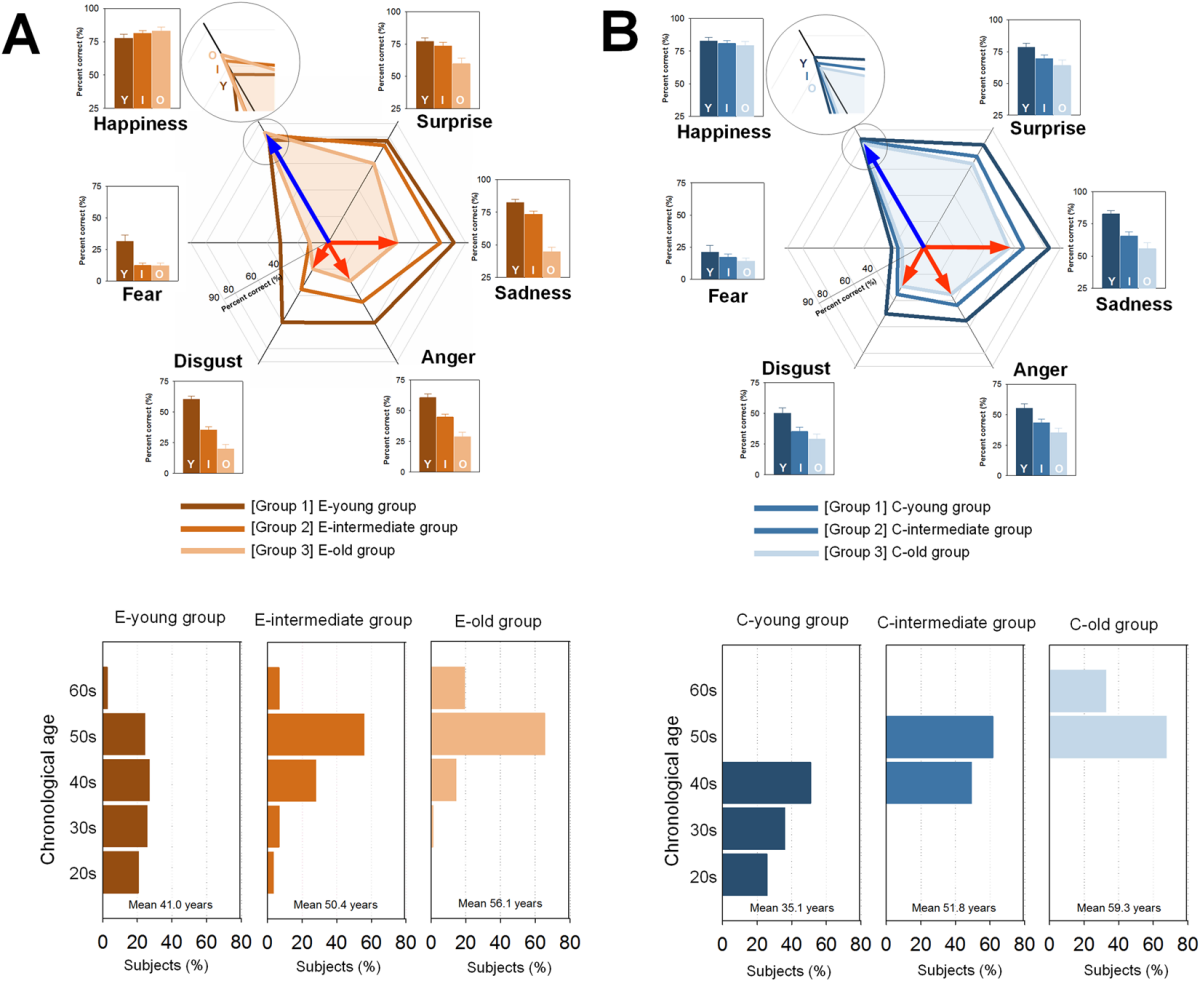
Distribution of performance on the Emotion Recognition Task (ERT)(upper) and chronological age (lower) among the groups according to emotional (**A**) and chronological (**B**) age grouping strategies. The E-intermediate and E-old groups outperformed the E-young group in happiness recognition. Performance on recognition of other emotions was lower in the E-intermediate and E-old groups relative to the E-young group. A similar pattern of performance on emotion recognition was observed among the chronological age groups. However, there was no difference in performance on happiness recognition among the chronological age groups. Significant group-differences in chronological age were observed among the emotional age groups as well as the chronological age groups. Y, I, and O of the bar graphs represent the performance on the ERT of the “emotionally or chronologically young”, “emotionally or chronologically intermediate-age” and “emotionally or chronologically old” groups, respectively.

The first group consisted of 78 individuals who performed above average in recognizing all six emotions. The second group of 122 subjects was characterized as chronologically older in age while having lower performance on recognition of negative emotions which are sadness, anger, disgust, and fear, as compared with the first group. However, performance on happiness recognition was higher in the second group relative to the first group. Subjects in the third group (n = 86) were chronologically older and performed poorly on recognition of all emotions except happiness relative to the first group. Likewise, the third group outperformed the first group in happiness recognition. Given these characteristics, the three groups were designated hereafter as the emotionally young (E-young), emotionally intermediate-age (E-intermediate), and emotionally old (E-old) groups, respectively (Table 1 and Fig. 1). The ratios between the mean correct identification of positive (happiness) and negative (sadness, anger, and disgust) emotions in the E-young, E-intermediate, and E-old groups were 1.16, 1.62, and 2.86, respectively. The blue arrows in Fig. 1A reflect the E-old group’s performance on happiness recognition while red arrows in Fig. 1A reflect the E-old group’s performance on recognition of negative emotions such as sadness, anger, and disgust. The correct identification rate of positive emotion was more than two-folds greater than that of negative emotions in the E-old group, while the correct identification rates between positive and negative emotions were similar in the E-young group.

**Table 1:** Characteristics of emotional and chronological age groups.

	Total (n = 286)	Empirically-derived grouping*			Grouping according to chronological age		
		Emotionally young group (n = 78)	Emotionally intermediate-age group (n = 122)	Emotionally old group (n = 86)	Chronologically young group (n = 78)	Chronologically intermediate-age group (n = 122)	Chronologically old group (n = 86)
Age, y	49.5 (10.4)	41.0 (12.1)	50.4 (8.4)	56.0 (4.5)	35.1 (7.3)	51.9 (3.0)	59.3 (2.3)
Age group, no (%)							
20–29 y	20 (7.0)	16 (20.5)	4 (3.3)	0 (0.0)	20 (25.6)	0 (0.0)	0 (0.0)
30–39 y	28 (9.8)	20 (25.6)	8 (6.6)	0 (0.0)	28 (35.9)	0 (0.0)	0 (0.0)
40–49 y	59 (20.6)	21 (26.9)	34 (27.9)	4 (4.7)	30 (38.5)	29 (23.8)	0 (0.0)
50–59 y	151 (52.8)	19 (24.4)	68 (55.7)	64 (74.4)	0 (0.0)	93 (76.2)	58 (67.4)
60–65 y	28 (9.8)	2 (2.6)	8 (6.6)	18 (20.9)	0 (0.0)	0 (0.0)	28 (32.6)
Male sex, no (%)	55 (19.2)	16 (20.5)	21 (17.2)	18 (20.9)	13 (16.7)	18 (14.8)	24 (27.9)
Education, y	14.6 (2.6)	15.0 (2.8)	14.5 (2.6)	14.3 (2.3)	14.8 (2.4)	14.9 (2.2)	14.0 (3.1)

All values are represented as means (SDs) unless indicated otherwise.

*Empirically derived groups were defined based on the results of cluster analysis on the emotion recognition task.

There were significant age differences between the E-young and E-intermediate groups (mean age of 41.0 years vs. 50.4 years, *P*_Bonferroni-corrected_ < 0.05, ES = 0.90) as well as between the E-young and E-old groups (mean age of 41.0 years vs. 56.0 years, *P*_Bonferroni-corrected_ < 0.05, ES = 1.44) (Fig. 1A).

We also evaluated the distribution of ERT performance according to the subjects’ chronological age. Subjects were classified according to chorological age into three groups, each of which was identical in sample size to its respective emotional age group (chronologically young [C-young] group, n = 78, mean ± SD, 35.1 ± 7.3 years old; chronologically intermediate-age [C-intermediate] group, n = 122, 51.9 ± 3.0; chronologically old [C-old] group, n = 86, 59.3 ± 2.3) (Table 1). All analyses were repeated using this chronological age grouping scheme and the results are shown in Fig. 1B. There were no differences in performance on the recognition of happy and fearful faces among the chronological age groups. However, task performance on the recognition of emotions other than happiness and fear decreased with increasing chronological age. Detailed information on the statistical values of the analyses according to the chronological age grouping scheme is presented in the Supplementary Results.

### Differences in intra-network functional connectivity among the emotional age groups

**Figure 2:**
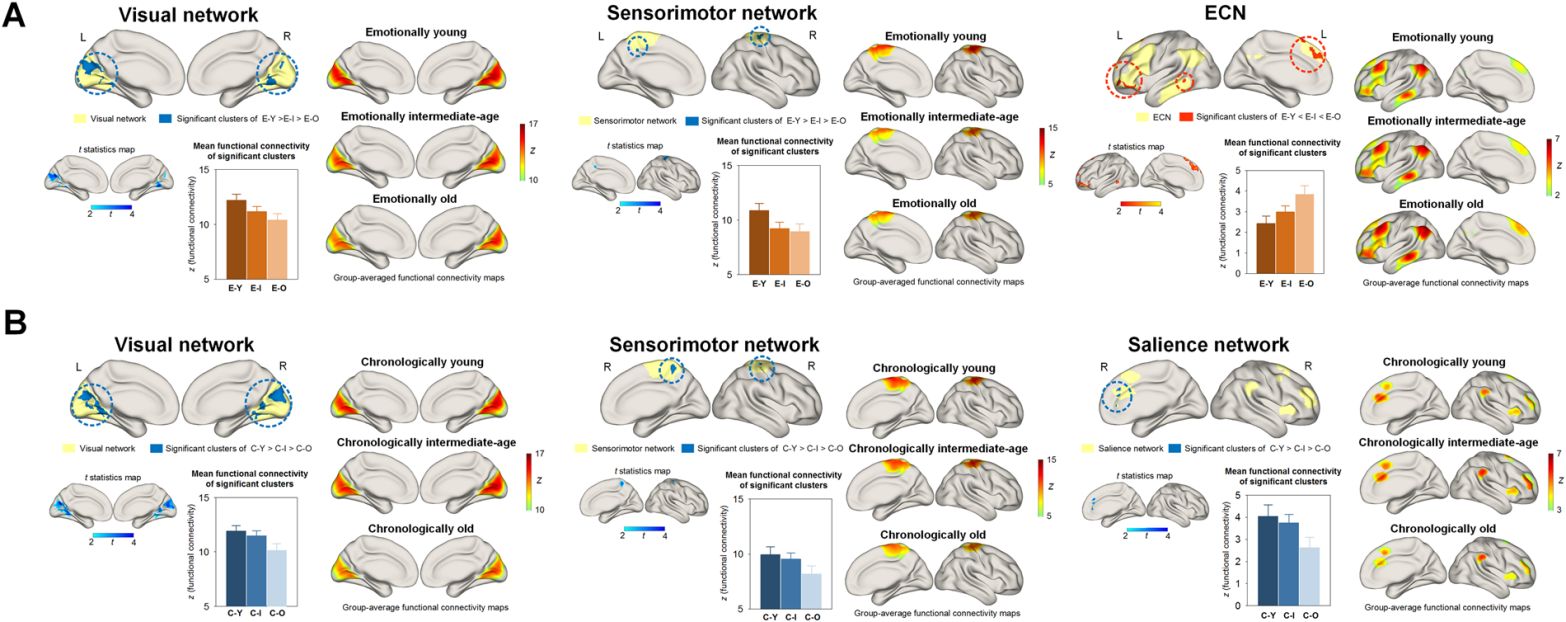
Significant clusters and *t*-statistic images for linear trends of intra-network functional connectivity among the emotional age groups (**A**) as well as the chronological age groups (**B**). Bar graphs represent the mean intra-network functional connectivity of significant clusters in each group. Error bars indicate 95% confidence intervals. A linear trend of decreased intra-network functional connectivity among the E-young, E-intermediate, and E-old groups was observed in the visual and sensorimotor networks at TFCE-corrected *P* < 0.05. In contrast, intra-network functional connectivity of the ECN increased linearly among the E-young, E-intermediate, and E-old groups. Among the chronological age groups, a significant decreasing trend in intra-network functional connectivity that is chronological age-dependent was found in the visual, sensorimotor, and salience networks. Detailed information on significant clusters is presented in Supplementary Figure 2, Supplementary Tables 1 and 2. Using BrainNet viewer^41^, three-dimensional rendering of RSNs was generated in the brain of standard space. Abbreviations: ECN, executive control network; TFCE, threshold-free cluster enhancement; RSN, resting state network; E-Y, emotionally young group; E-I, emotionally intermediate-age group; E-O, emotionally old group; C-Y, chronologically young group; C-I, chronologically intermediate-age group; C-O, chronologically old group.

We found that clusters showing a significant linear decreasing trend in intra-network functional connectivity from the E-young, to the E-intermediate, and to E-old groups were located in the visual and sensorimotor networks at corrected *P* < 0.05 (Fig. 2A, Supplementary Figure 2, and Supplementary Table 1). In contrast, clusters showing a significant linear increasing trend in intra-network functional connectivity from the E-young, to the E-intermediate, and to the E-old groups were found in the executive control network (ECN) at corrected *P* < 0.05 (Fig. 2A, Supplementary Figure 2, and Supplementary Table 1). There were no significant differences in intra-network functional connectivity of the default mode network (DMN) and salience network among the emotional age groups.

We also examined whether intra-network functional connectivity of major RSNs differ according to the subjects’ chronological age. Chronological aging-related decreases in the intra-network functional connectivity were observed in the visual and sensorimotor networks (C-young > C-intermediate > C-old groups, Fig. 2B, Supplementary Figure 2, and Supplementary Table 2) as well as in the salience network (Fig. 2B, Supplementary Figure 2, and Supplementary Table 2).

### Differences in inter-network functional connections among emotional age groups

**Figure 3:**
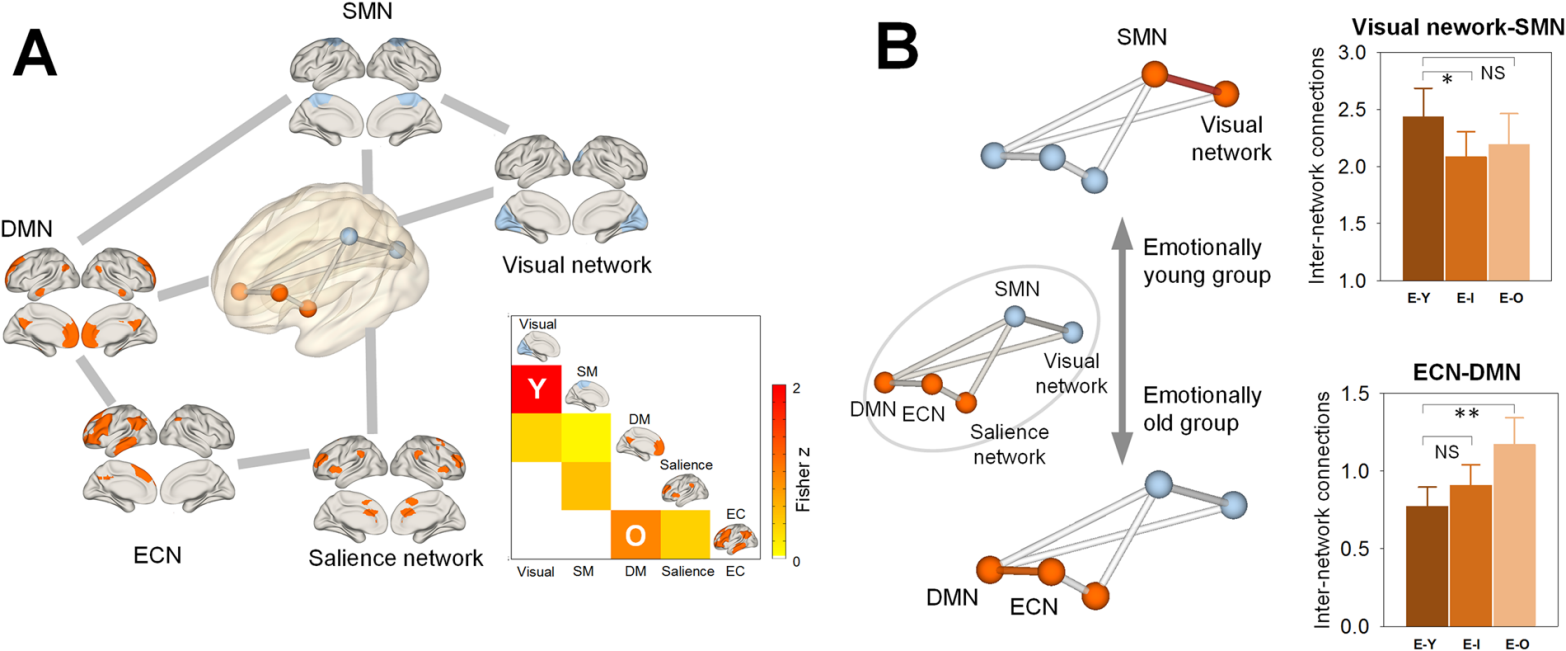
Inter-network correlation matrix for the whole group of participants (**A**) and differences in inter-network connectivity strength between the emotional age groups (**B**). Significant group differences in inter-network connectivity strength are shown as red lines in panel B. Using the Fisher *r* to *z* transformation, inter-network correlation scores were converted to z scores. Decreased inter-network connectivity between the visual and somatosensory networks was observed in the E-intermediate group relative to the E-young group (Y in the correlation matrix of panel A). In contrast, inter-network functional connections between the ECN and DMN was greater in the E-old group as compared to the E-young group (O in the correlation matrix of the panel A).*Permutation-adjusted *P* < 0.05; **Permutation-adjusted *P* < 0.01. Abbreviations: SMN, sensorimotor network; DMN, default mode network; ECN, executive control network; E-Y, emotionally young group; E-I, emotionally intermediate-age group; E-O, emotionally old group; NS, non-significant.

For the estimation of inter-network connections, subject-wise correlation matrices each consisting of 5 network nodes (visual, sensorimotor, DMN, salience, and ECN) were computed based on regularized partial correlation analysis (Fig. 3A).

The E-intermediate group had lower inter-network functional connections between the visual and sensorimotor networks than the E-young group (*P* ^*PA*^ = 0.02, ES = 0.29, Fig. 3B). In contrast, the magnitude of inter-network functional connections between the ECN and DMN was greater in the E-old group relative to the E-young group (*P* ^*PA*^ = 0.001, ES = 0.55, Fig. 3B). There were no differences in inter-network connections between any of the RSNs among the chronological age groups.

In an effort to provide evidence supporting the phenomenon and construct of emotional aging and its distinct differentiation from that of chronological aging, we have performed two additional analyses, each of which was based on an age-restricted subsample. Repeated analyses were performed for participants between the age 50 and 59 (n = 151, mean age [SD] = 55.0 [2.8] years) as well as for those between the age 40 and 49 (n = 59, mean age [SD] = 45.1 [2.8] years). Although the level of statistical significance slightly changed with decreasing sample size, these repeated analyses demonstrated a similar clinical and network pattern of emotional aging to those obtained from all participants without the age-range restriction. Furthermore, we did not find significant differences in chronological age between the emotional age subgroups in these sensitivity analyses. The detailed information for these sensitivity analyses is presented in the Supplementary Results and Supplementary Figure 3.

## Discussion

The current study is the first to identify the neural correlates of emotional aging from a brain network perspective. Along the process of emotional aging, performance on recognition of happiness improves, while that of other negative emotions decline. At a brain network perspective, the intra-network functional connectivity of the visual and sensorimotor networks decreased with emotional and chronological aging, and that of ECN increased with emotional, but not with chronological, aging. Furthermore, the inter-network functional connections between the ECN and DMN also increased with emotional aging, potentially suggesting an enhanced top-down integration of self-referential information during emotional processing. Taken together, the present study suggests that different strategies involving the ECN are utilized for emotion recognition in relation to emotional aging.

Interestingly, we found that intra-network functional connectivity of the ECN has a strong tendency to increase across emotional age groups, from the direction of the E-young to E-intermediate, and then to E-old groups. This, along with the absence of this trend in the chronological age groups, suggests that changes in functional connectivity of the ECN are closely associated with emotional aging than aging per se. While perceptual processing of visual and sensory domains reduces with chronological aging^6^, more central and higher cognitive functional involvement may compensate for these aging-related decline in brain perceptual performance^6,15–17^.

Previous studies have reported that older adults tend to focus more on positive emotions rather than negative emotions^17–19^. One explanation behind this statement is that arousal responses to negative stimuli are reduced, due to attenuated brain functions and general cognitive decline of the aging brain^5^. Older people may feel negative emotions in a less potent manner due to decreased transmission of emotional stimuli from the external world^2^. Interestingly, the current study supports this theory, as we found that intra-network functional connectivity of the primary sensory networks was reduced in the emotionally older groups relative to the emotionally young as well as in the chronologically older groups relative to the chronologically young.

An alternative explanation is that the recruitment of different regulatory strategies may contribute to changes in emotional processing with advancing age^2,4^. For instance, older adults seem to use the prefrontal cortex, which subserves emotion suppression strategies during recognition of negative emotions^11–13,18,20^. Our findings support this hypothesis, by providing evidence that stronger inter-network functional connections are present between the ECN and DMN in the emotionally older groups relative to the emotionally young group.

Recent studies have reported that the DMN, along with other task-relevant brain networks such as the ECN, are involved in autobiographical tasks^10,21^. Interestingly, the capacity of the DMN for processing the self-referential information seems relatively preserved or even increased during the encoding of positive information, compared to negative, in healthy aging^22,23^. Furthermore, the inter-network functional connections between the DMN and ECN during autobiographical as well as visuospatial tasks were also greater in old adults than in young adults^24^.

Considering this expanded role of the DMN, it is possible that emotionally old individuals integrate situational and self-referential information processing more efficiently by enhancing the inter-network connections between the DMN and ECN, therefore showing positive outcomes in emotion regulation^24^. This idea can be supported by previous studies, which reported that inter-network connections between the DMN and ECN were lower in subjects with depression, but enhanced in mindfulness practitioners^24,25^. Thus, the increased inter-network functional connections of the DMN and ECN observed in emotionally older individuals may facilitate focused internal attention, top-down evaluation of internal information, and integration of self-referential information during emotion recognition and regulation^26^.

As expected, decreased intra-network functional connectivity of the salience network in the chronologically older subjects was observed, as these findings are generally consistent with previous studies^27,28^. However, there were no differences in intra-network functional connectivity of the DMN among the emotional nor chronological age groups. The DMN has received substantial attention in the field of research regarding the aging brain since it includes important brain regions related to Alzheimer’s disease, such as the posterior cingulate cortex and hippocampus^29^. Although studies often report that intra-network functional connectivity of the DMN decrease in association with aging^28^, there are other studies that claim there are no significant changes, as in the current study, presumably due to the variations of sample characteristics^28,30^. For example, DMN connectivity decrease was significant in comparisons of young and elderly adults but not that of middle-aged and elderly adults^28,30^. Thus, the absence of DMN connectivity decrease with chronological aging may be partially due to the sample characteristics that were not geared to detect such alterations.

Since there is no criterion to define the extent of emotional aging, cluster analysis was used in the present study to classify a large sample of participants aged between 20 and 65 years into the empirically driven groups based on their ERT performance. To validate this data-driven grouping strategy, we repeated analyses for groups classified by their chronological age. A similar pattern was observed, even though results from the emotional age groups based on the cluster analysis of ERT performance may better reflect the characteristics of emotional aging. Interestingly, a small portion of participants who were 50 years and older (n = 21, 12% of total participants with age ≥ 50 years old) was classified into the E-young group. There were no demographic differences between these 21 chronologically “old” but emotionally young subjects and the remaining 158 chronologically old and also emotionally old subjects (age, *t* = 0.28, *P* = 0.78; sex, *χ* ^2^ = 0.02, *P* = 0.90). It would be interesting to evaluate the neural mechanisms for this grouping in future work with a larger and more balanced sample size.

Limitations of the study include the following. A cultural perspective should be taken into consideration in interpreting our findings given the cultural differences in brain activity with regards to social affective processes^31^. Considering that cross-sectional correlational studies using inter-network connections, as in our study, cannot provide directional information of brain networks, the directionality of the observed hyperconnectivity between the ENC and DMN in the emotionally older group is yet to be determined. As such, whether this hyperconnectivity reflects a strengthened prefronto-parietal control over the DMN or vice versa, or both, is unknown. It should be noted that an empirically derived grouping strategy using information regarding the performance on emotion recognition has been applied in the current study to determine the characteristics of emotional aging, since there has yet to be a definite criterion for the extent of emotional aging. Therefore, the current results should be interpreted with caution to avoid recursive use of the findings. As such, the clinical and network characteristics of emotional age groups, which have been presented in the present proof-of-concept study, should be validated and replicated in future studies using a predefined clinical definition of emotional aging.

In the present study, repeated analyses in relatively age-range restricted subsamples yielded similar findings to those from all participants. These findings have provided preliminary evidence suggesting that people who are similar in their chronological age can vary in emotional age. However, in order to make a clear distinction between the construct of emotional and chronological aging, the current findings should be independently replicated in a strictly age-restricted sample, such as, in participants who are all identical in age while having different characteristics related to emotional aging.

In conclusion, the current proof-of-concept study provides evidence which supports the presence of specific brain network correlates underlying emotional aging. We found that the inter-network functional connections between ECN and DMN as well as intra-network functional connectivity of the ECN were enhanced with emotional aging. These findings may explain the behavioral observation that the recognition of positive affect tends to enhance while that of negative emotions decline with emotional aging.

## Materials and Methods

### Participants and Task Procedures

Two hundred eighty-six healthy adults (mean age = 49.5 years, 55 males, age range = 20–65 years) who had no history of neurological and psychiatric disorders. All participants had normal or corrected-to-normal visual acuity. The study protocol was approved by the Bioethics Committee of Ewha W. University and all participants provided written informed consent prior to participation. All procedures and methods were performed in accordance with institutional and national guidelines and regulations.

Emotion recognition was measured using the ERT implemented in the Cambridge Neuropsychological Test Automated Battery (http://www.camcog.com). This task is a computer-based paradigm to assess the recognition accuracy of six basic emotions including happiness, surprise, sadness, anger, disgust, and fear. Two blocks of 15 stimuli for each facial emotion with different levels of intensity (90 stimuli for each block) were randomly displayed to participants. After a 200 milliseconds display of facial emotion, participants were asked to decide which emotion the face is depicting, by choosing among the six emotional labels on the screen. Percentage of correct answers for each emotion was calculated as outcome measures for performance of emotion recognition.

### Classification of Participants

As a data-driven empirical approach for the classification of participants, a two-step approach which includes the hierarchical agglomerative cluster analysis to identify the number of distinct clusters, followed by the k-means cluster analysis to the characteristics of the distinct profiles, was performed on participants using percent correct measures for each emotion recognition^32^. In order to determine the best-distinguished cluster solution for this case, the hierarchical agglomerative cluster analysis using Ward’s method of minimum variance with a squared Euclidean distance measures^33^ was applied. By examining the dendrogram, which displayed how each individual was categorized, it became apparent that a three-cluster solution was appropriate. Based on this hierarchical solution, the data was then entered into the k-means cluster analysis with a three-cluster option to determine whether the clusters could be theoretically interpreted as well as consist of at least 20% of all participants. The robustness of the cluster analysis was validated using a confirmatory discriminant function analysis to evaluate the classification accuracy of the final cluster solution. A leave-one-out cross-validation was implemented to assess the generalization of the predictive reliability of the clusters.

### Functional MRI Data Acquisition and Processing

High-resolution structural and functional imaging was performed using a 3.0 Tesla Philips Achieva MR scanner (Philips Medical System, Netherlands) equipped with a 32-channel head coil. Resting-state fMRI data were obtained with an echo planar imaging sequence [repetition time, 2,000 ms; echo time, 21 ms; flip angle, 76°; field of view, 220 × 220 mm^2^; slice thickness, 3.5 mm; 200 volumes; 38 slices]. During data acquisition, participants were instructed to think of nothing in particular, to keep eyes closed, and to stay awake. High-resolution structural images were also acquired for coregistration to the functional MRI data using a three-dimensional T1-weighted magnetization-prepared rapid gradient echo imaging sequence with the following acquisition parameters: repetition time, 7.4 ms; echo time, 3.4 ms; flip angle, 8°; field of view, 220 × 220 mm^2^; slice thickness, 1 mm; 180 contiguous sagittal slices.

Functional data preprocessing was carried out using the FMRIB Software Library tools (FSL, http://www.fmrib.ox.ac.uk/fsl). A brief description for the standard preprocessing steps is as follows: motion correction using multi-resolution rigid body co-registration^34^, the removal of non-brain tissues, spatial smoothing with a Gaussian kernel of full width at half maximum of 5.0 mm, and high-pass filtering at 0.01 Hz. Functional images were first co-registered to the corresponding high-resolution T1-weighted images and further registered to the standard Montreal Neurological Institute (MNI) 152 template using a 12-parameter affine transformation algorithm. Structural artifacts were removed from each data set using single-subject ICA with automatic dimensionality estimation using Multivariate Exploratory Linear Optimized Decomposition into Independent Components (MELODIC)^35,36^ followed by FBRIB’s ICA-based Xnoiseifier (FIX)^37^.

The resultant preprocessed images were decomposed into a set of independent one-dimensional time series and related three-dimensional spatial maps. Group ICA, a model-free and data-driven approach, was applied to this process implemented in the FSL^35,36^. The component number was set to 25 with a temporal concatenation approach. Using the resting-state fMRI data of 286 participants, group independent components were classified into RSNs or artifacts by the visual inspection of an experienced investigator (S.Y.). The standard for classifying the data as meaningful RSNs was based on the anatomically and functionally classical RSNs previously described^36^. Among the thirteen RSNs identified, five RSNs of interest including the visual, sensorimotor, default mode, salience, and executive control networks were chosen in subsequent analyses. Component information and spatial maps of all available components, which demonstrated considerable correspondence to major RSNs and were thresholded at z = 4.3 (*P* = 0.0001), are presented in Supplementary Figure 1.

In order to investigate intra-network functional connectivity among participants as well as time points, a dual linear regression approach was used to estimate subject-specific time courses and spatial maps corresponding to the predefined five RSNs of interests^35^. As the first stage of dual regression, spatial regression was performed to fit the group ICA maps against each subject’s functional data and create the average subject-specific time series for the RSNs of interest. In addition, temporal regression was performed against the subjects’ functional data to create the subject-specific spatial maps for the RSNs of interest as the output of the second stage of dual regression.

Inter-network functional connections were estimated based on individual time series of RSNs of interest using the FSLNets (http://fsl.fmrib.ox.ac.uk/fsl/fslwiki/FSLNets). The subject-wise correlation matrices were generated from a regularized partial correlation between all pairs of time series of the RSNs of interest (5-by-5 matrices of 286 subjects). Correlation coefficients were transformed to z-scores using Fisher’s z transformation and corrected for temporal autocorrelation, then extracted from each subject for subsequent analyses.

### Statistical Analyses

In order to examine whether intra-network connectivity may differ according to emotional aging, a linear trend of increased or decreased voxel-wise functional connectivity among the E-young, E-intermediate, and E-old groups was fitted on the spatial maps for each RSN of interest using the general linear model. A non-parametric permutation test (5,000 permutations)^38^ with threshold-free cluster enhancement (TFCE) using a significance threshold of *P* < 0.05 and a minimal cluster size of 5 voxels was implemented to perform a family-wise error correction for multiple comparisons^39^. *Z* values representing connectivity of the corresponding RSNs were extracted from significant clusters of voxels from each subject’s spatial map and the mean connectivity values of significant clusters are visualized in bar graphs (Fig. 2).

Inter-network connections were also compared among the emotional age groups as well as the chronological age groups using the robust regression analysis. Group membership (young, intermediate, or old groups) was introduced as a dummy variable to obtain the statistically significant values of inter-network connections when compared with the reference group. Emotionally or chronologically young groups, as defined according to the emotional or chronological age grouping strategy, respectively, was the reference group for these analyses. To account for multiple comparisons, the group memberships of the 286 subjects were randomly permutated 5,000 times to obtain an empirical null distribution of effects under the null-hypothesis. A permutation-adjusted *P* value was computed based on the proportion of permutations with *P* values that were greater than the observed values from the actual data set, while being under the 5,000 simulated null distributions^40^.

Two-tailed significance of *P* < 0.05 was considered to be statistically significant. Cohen’s d was estimated to measure effect size. Data were analyzed using Stata SE, v11.0 (Stata Corp, College Station, TX).

## Electronic supplementary material

Supplementary Material
