# Supplementary material for: Brain Network Correlates of Emotional Aging
Source: Sci Rep. 2017 Nov 14;7:15576. doi: 10.1038/s41598-017-15572-6 (PMC5686193; doi:10.1038/s41598-017-15572-6)
Supplement: Supplementary file 1 — Supplementary Material [file 41598_2017_15572_MOESM1_ESM.doc]

**Supplementary Information**

**Title**

Brain Network Correlates of Emotional Aging

**Authors**

Youngwook Lyoo^1^ and Sujung Yoon^2*^

**Author Affiliations**

^1^Seoul National University College of Medicine, Seoul, South Korea

^2^Ewha Brain Institute, Ewha Womans University, Seoul, South Korea

*correspondence and requests for materials should be addressed to S. Y.

(email: sujungjyoon@ewha.ac.kr).

**Supplementary Results**

*Differences in ERT performance among chronological age groups*

Significant reductions in task performance on recognition of surprise, sadness, anger, and disgust were observed between the C-young and C-intermediate groups (surprise, *P*_Bonferroni-corrected_ < 0.05, ES = 0.50; sadness, *P*_Bonferroni-corrected_ < 0.05, ES = 0.84; anger, *P*_Bonferroni-corrected_ < 0.05, ES = 0.65; disgust, *P*_Bonferroni-corrected_ < 0.05, ES = 0.69) as well as between the C-young and C-old groups (surprise, *P*_Bonferroni-corrected_ < 0.05, ES = 0.82; sadness, *P*_Bonferroni-corrected_ < 0.05, ES = 1.32; anger, *P*_Bonferroni-corrected_ < 0.05, ES = 1.10; disgust, *P*_Bonferroni-corrected_ < 0.05, ES = 0.98)(Figure 1B). There were no differences in performance on recognition of happy and fearful faces between the C-young and C-intermediate groups (happiness, *P*_Bonferroni-corrected_ > 0.05, ES = 0.16; fear, *P*_Bonferroni-corrected_ > 0.05, ES = 0.24), nor between the C-young and C-old groups (happiness, *P*_Bonferroni-corrected_ > 0.05, ES = 0.28; fear, *P*_Bonferroni-corrected_ > 0.05, ES = 0.42).

*Sensitivity analyses in two age-restricted subsamples (Supplementary Figure 3)*

*Grouping and chronological age:* Two sensitivity analyses were performed, each of which used an age range-restricted subsample. Sensitivity analysis 1 included individuals between the age 50 and 59 (n = 151, mean age [SD] = 55.0 [2.8] years) and sensitivity analysis 2 included individuals between the age 40 and 49 (n = 59, mean age [SD] = 45.1 [2.8] years). In sensitivity analysis 1, K-means cluster analysis yielded three distinct subgroups based on percent correct measures for each emotion recognition. The E-young, E-intermediate, and E-old subgroups included 38 (mean age [SD] = 54.7 [2.8] years), 70 (mean age [SD] = 54.8 [3.0] years), and 43 (mean age [SD] = 55.5 [2.4] years) individuals, respectively. There was no significant difference in chronological age between the subgroups (*β* = 0.10, *P* = 0.22). In sensitivity analysis 2, a two-cluster solution derived through K-means cluster analysis was used due to a relatively small sample size. Fifty-nine individuals in their forties were divided into the two groups including the E-young (n = 26, mean age [SD] = 45.7 [2.8] years) and E-old (n = 33, mean age [SD] = 44.6 [2.8] years) subgroups. These subgroups were similar in chronological age (*β* = -0.20, *P* = 0.13).

*Group comparisons of ERT performance*: Robust regression analysis was used to determine differences in the ERT performance level among these emotional age subgroups in sensitivity analysis 1. The group membership was introduced as a dummy variable, with the E-young subgroup as the reference group. Compared to the E-young subgroup (n = 38), the E-intermediate (n = 70, *P* < 0.001) and E-old subgroups (n = 43, *P* < 0.001) had greater performance on happiness recognition. However, performance on negative emotion recognition were lower in the E-intermediate subgroup (sadness, *P* < 0.001; anger, *P* < 0.001; disgust, *P* < 0.001) and in the E-old subgroup (sadness, *P* < 0.001; anger, *P* < 0.001; disgust, *P* < 0.001), compared to the E-young subgroup. For the recognition of fear and surprise, the E-old subgroup had lower performance as compared to the E-young subgroup (fear, *P* < 0.001; surprise, *P* < 0.001), while no differences were found between the E-intermediate and E-young subgroups (fear, *P* = 0.06; surprise, *P* = 0.60).

In sensitivity analysis 2, a similar pattern of group differences in ERT performance was observed. The E-old subgroup had greater performance on happiness recognition than the E-young subgroup (*P* = 0.006). In contrast, lower performance on recognition of anger (*P* < 0.001), disgust (*P* < 0.001), and fear (*P* = 0.001) were observed in the E-old subgroup as compared to the E-young subgroup. There were no differences in performance on the recognition of surprise (*P* = 0.88) and sadness (*P* = 0.39) between the subgroups.

*Group comparisons in intra-network and inter-network functional connections:* Values of intra-network functional connectivity were extracted from clusters that showed a significant linear trend among the E-young, E-intermediate, and E-old groups. The values were then used in subsequent sensitivity analyses.

In sensitivity analysis 1, the values of intra-network functional connectivity extracted from the clusters of the executive control network (ECN) showed a significant linear increasing trend from the direction of E-young, to the E-intermediate, to the E-old subgroups (*β* = 0.21, *P* = 0.01). Inter-network functional connections between the ECN and default mode network (DMN) were greater in the E-old subgroup relative to the E-young subgroup (*P* = 0.08) with a marginal statistical significance.

In sensitivity analysis 2, intra-network functional connectivity of the ECN was greater in the E-old subgroup than in the E-young subgroup (*β* = 0.27, *P* = 0.04). Similarly, the E-old subgroup had enhanced inter-network functional connections between the ECN and DMN as compared to the E-old subgroup (*P* = 0.07), although this difference did not reach statistical significance.

| 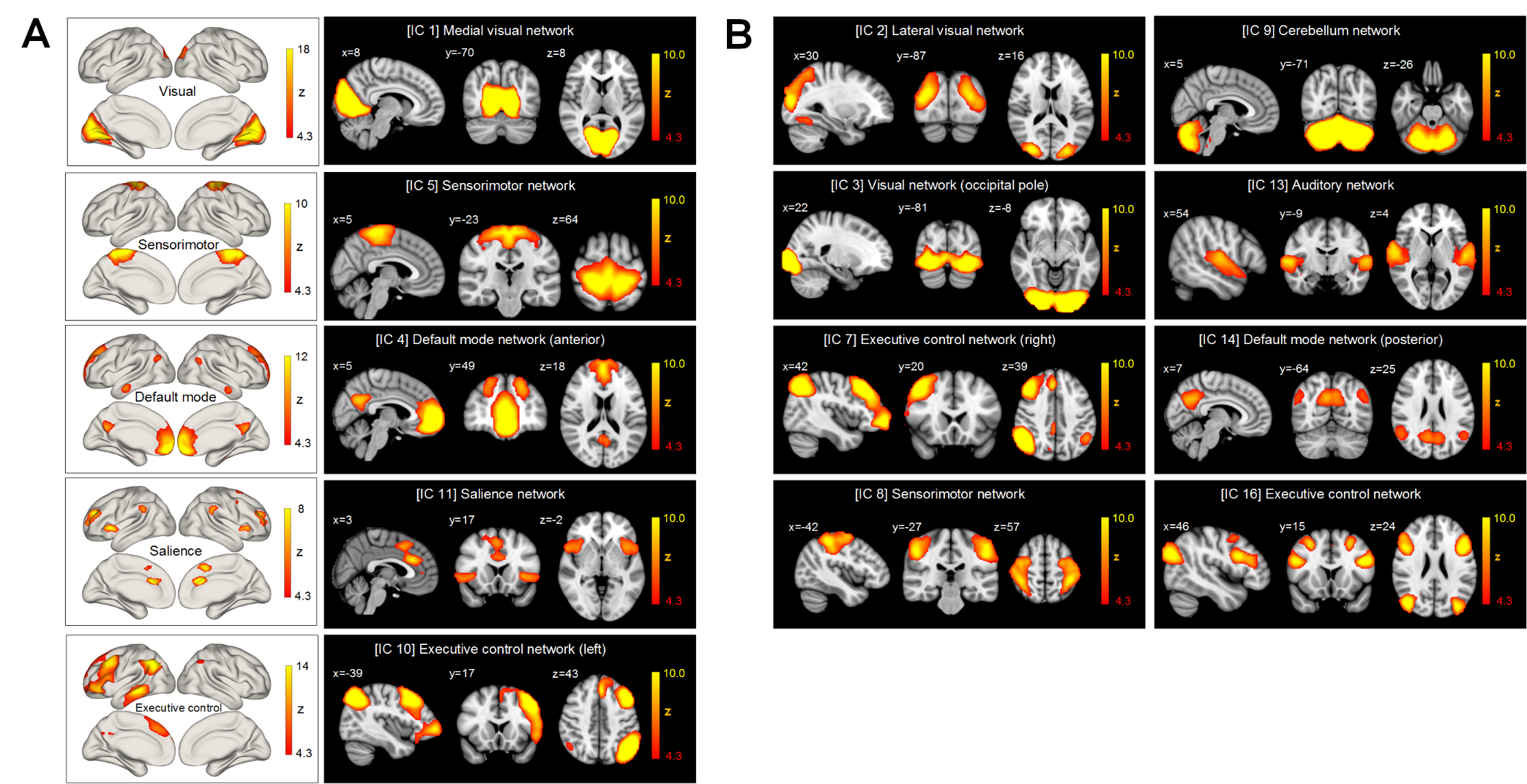 |
| --- |
| **Supplementary Figure 1.** Among 25 independent components (ICs) based on group ICA on 286 participants, five RSNs of interest including the visual, sensorimotor, default mode, salience and executive control networks were identified and were focused on in the present study (A). Spatial maps were converted to z score images and were thresholded at z = 4.3 (*P* = 0.0001). The number of IC indicates the amount of variance explained by the corresponding IC in decreasing order. Other ICs, which demonstrate considerable correspondence to major RSNs previously described, are presented in the panel B. |

| 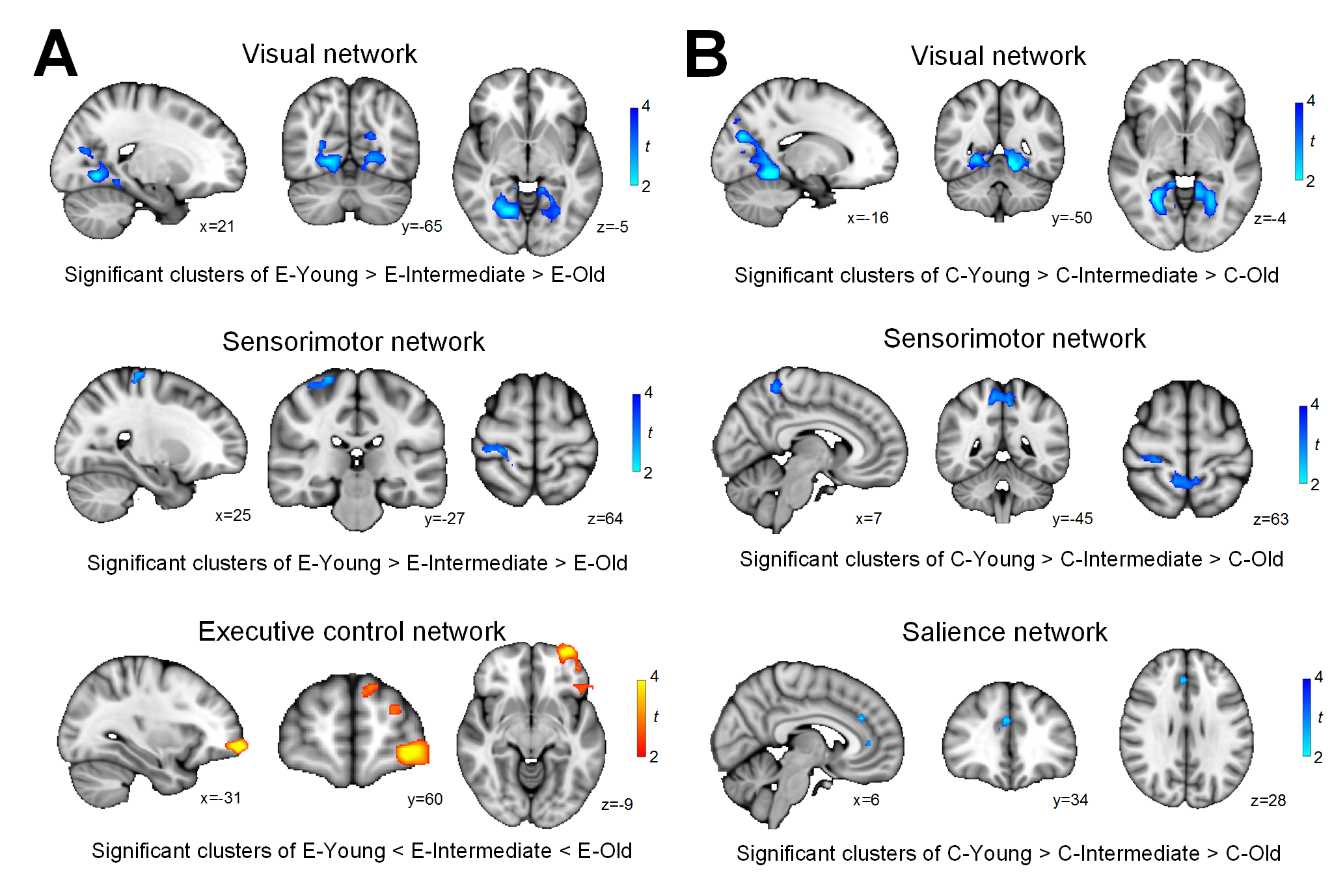 |
| --- |
| **Supplementary Figure 2.** *T*-statistic images for significant linear trends for decreased (in cold colors) or increased (in warm colors) functional connectivity among emotional (A) and chronological (B) age groups. |

| 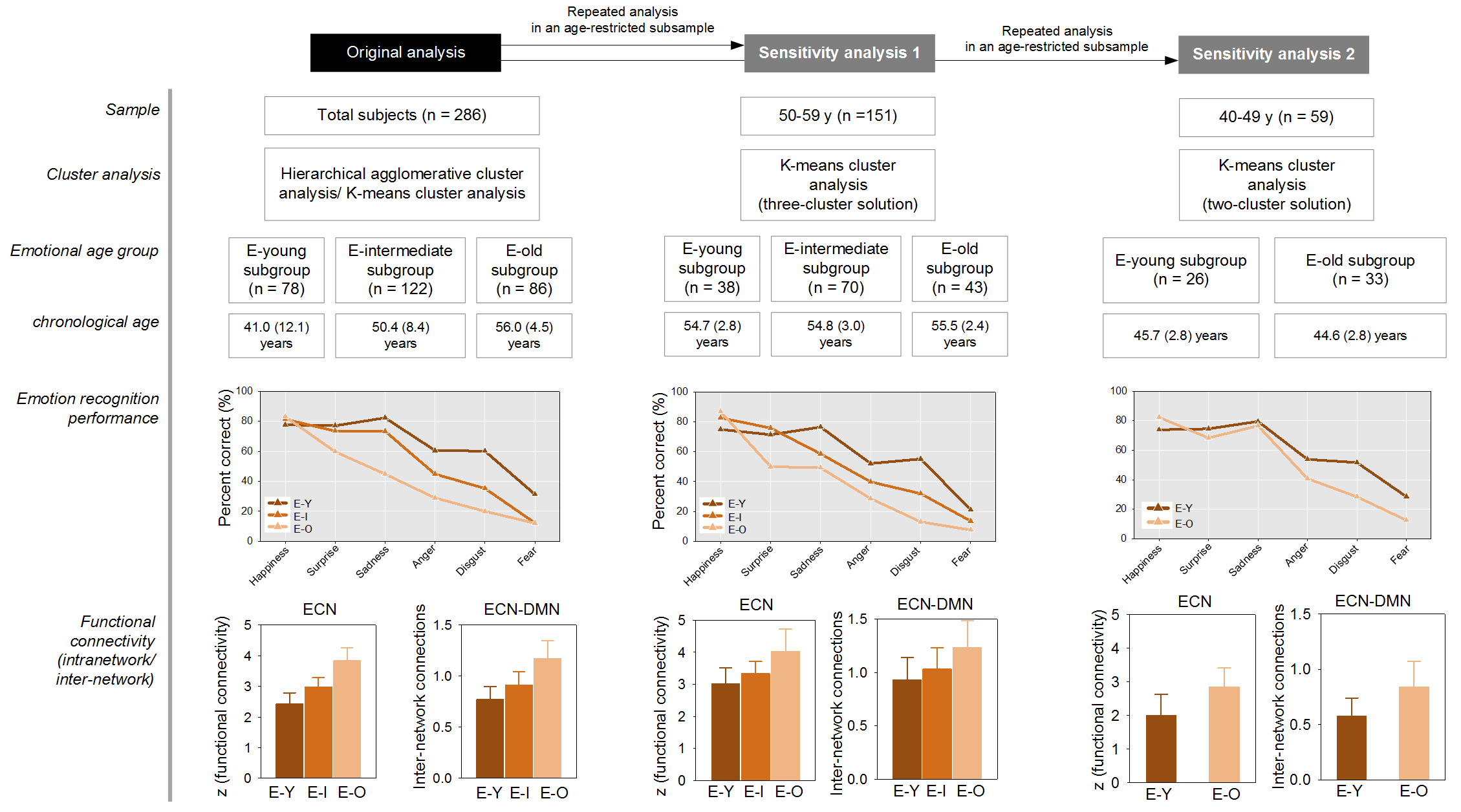 |
| --- |
| **Supplementary Figure 3.** Summary of results from the original analysis and two additional sensitivity analyses using age-restricted subsamples from total participants. Sensitivity analysis 1 included 151 participants in their fifties (mean age [SD] = 55.0 [2.8] years) and sensitivity analysis 2 included 59 participants in their forties (mean age [SD] = 45.1 [2.8] years). K-means cluster analysis in sensitivity analysis 1 yielded three distinct subgroups based on percent correct measures for each emotion recognition task. In contrast to the results from the original analysis, there were no differences in chronological age between these subgroups (*β* = 0.10, *P* = 0.22). However, similar results to those from the original analysis were obtained for the performance on emotional recognition tasks as well as functional connectivity. For sensitivity analysis 2, a two-cluster solution was derived through K-means cluster analysis due to the relatively small sample size (n = 59). There were no significant chronological age differences between the two emotional age groups (*β* = -0.20, *P* = 0.13). However, a similar pattern of performance on emotional recognition and functional connectivity was observed as compared to those from the original analysis. In summary, the current sensitivity analyses have provided the preliminary evidence supporting that emotional aging may be a distinct construct from chronological aging. |

| **Supplementary Table 1.** Cluster information of voxel-wise functional connectivity alterations related to the extent of emotional aging (also refer to Figure 2A). | | | | | | | | |
| --- | --- | --- | --- | --- | --- | --- | --- | --- |
| Network | Anatomical location | Cluster size (mm^3^) | Maximum *t* value |  | MNI atlas coordinates | | |  |
|  |  |  |  |  | (location of maximum *t*-value) | | |  |
|  |  |  |  |  | *x* | *y* | *z* |  |
| Decreases in functional connectivity among the groups (E-young > E-intermediate > E-old groups) | | | | | | | | |
| Visual | Lingual gyrus | 34,752 | 5.04 |  | -18 | -46 | -12 |  |
| Sensorimotor | Postcentral gyrus | 3,840 | 3.74 |  | 22 | -30 | 68 |  |
|  | Precentral gyrus | 1,152 | 2.56 |  | -6 | 42 | 44 |  |
| Increases in functional connectivity among the groups (E-young < E-intermediate < E-old groups) | | | | | | | | |
| ECN | Frontal pole | 22,656 | 4.98 |  | -30 | 62 | -8 |  |
|  | Middle temporal gyrus | 1,216 | 3.09 |  | -66 | -46 | -8 |  |
| The general linear model was used to define clusters of a significant linear trend for decreased or increased functional connectivity among the E-young, E-intermediate, and E-old groups. Regions of significant alterations in functional connectivity at a TFCE-corrected *P*< 0.05 were defined as the clusters.  Abbreviations: MNI, Montreal Neurological Institute; ECN, executive control network; L, left; R, right; TFCE, threshold-free cluster enhancement. | | | | | | | | |

| **Supplementary Table 2.** Cluster information of voxel-wise functional connectivity alterations related to the extent of chronological aging (also refer to Figure 2B). | | | | | | | | |
| --- | --- | --- | --- | --- | --- | --- | --- | --- |
| Network | Anatomical location | Cluster size (mm^3^) | Maximum *t* value |  | MNI atlas coordinates | | |  |
|  |  |  |  |  | (location of maximum *t*-value) | | |  |
|  |  |  |  |  | *x* | *y* | *z* |  |
| Decreases in functional connectivity among the groups (C-young > C-intermediate > C-old groups) | | | | | | | | |
| Visual | Lingual gyrus | 52,224 | 4.64 |  | -14 | -50 | -12 |  |
| Sensorimotor | Postcentral gyrus | 9,664 | 3.58 |  | -10 | -42 | 52 |  |
| Salience | Paracingulate gyrus | 2,304 | 3.92 |  | 6 | 34 | 28 |  |
| The general linear model was used to define clusters of a significant linear trend for decreased functional connectivity among the C-young, C-intermediate, and C-old groups. Regions of significant alterations in functional connectivity at a TFCE-corrected *P*< 0.05 were defined as the clusters.  Abbreviations: MNI, Montreal Neurological Institute; L, left; R, right; TFCE, threshold-free cluster enhancement. | | | | | | | | |
